# Receptor tyrosine phosphatase PTPγ is a regulator of spinal cord neurogenesis

**DOI:** 10.1016/j.mcn.2010.11.012

**Published:** 2011-02

**Authors:** Hamid Hashemi, Michael Hurley, Anna Gibson, Veera Panova, Viktoria Tchetchelnitski, Alastair Barr, Andrew W. Stoker

**Affiliations:** a Neural Development Unit, UCL Institute of Child Health, University College London, 30 Guilford Street, London WC1N 1EH, UK; b Structural Genomics Consortium, University of Oxford, Old Road Campus Research Bldg., Roosevelt Drive, Headington, Oxford OX3 7DQ, UK

**Keywords:** RPTP, Receptor protein tyrosine phosphatase, RTK, Receptor tyrosine kinase, GOF, Gain-of-function, LOF, Loss-of-function, MN, Motor neuron, LMC, Lateral motor column, Protein tyrosine phosphatase, Motor neuron, Neural progenitor, Wnt/beta catenin, TCF, Spinal cord

## Abstract

During spinal cord development the proliferation, migration and survival of neural progenitors and precursors is tightly controlled, generating the fine spatial organisation of the cord. In order to understand better the control of these processes, we have examined the function of an orphan receptor protein tyrosine phosphatase (RPTP) PTPγ, in the developing chick spinal cord. Widespread expression of PTPγ occurs post-embryonic day 3 in the early cord and is consistent with a potential role in either neurogenesis or neuronal maturation. Using gain-of-function and loss-of-function approaches in ovo, we show that PTPγ perturbation significantly reduces progenitor proliferation rates and neuronal precursor numbers, resulting in hypoplasia of the neuroepithelium. PTPγ gain-of-function causes widespread suppression of Wnt/β-catenin-driven TCF signalling. One potential target of PTPγ may therefore be β-catenin itself, since PTPγ can dephosphorylate it in vitro, but alternative targets are also likely. PTPγ loss-of-function is not sufficient to alter TCF signalling. Instead, loss-of-function leads to increased apoptosis and defective cell–cell adhesion in progenitors and precursors. Furthermore, motor neuron precursor migration is specifically defective. PTPγ therefore regulates neurogenesis during a window of spinal cord development, with molecular targets most likely related to Wnt/β-catenin signalling, cell survival and cell adhesion.

## Introduction

The spinal cord is an excellent model system in which to study the temporospatial proliferation, migration and differentiation of neuronal progenitors (Poh et al., 2002). The pre-patterning of the cord dorsoventrally by morphogens lays down progenitor domains for interneurons and motor neurons (MNs) (Poh et al., 2002) and these progenitors then generate precursors that migrate laterally to their final positions, before differentiating (Shirasaki and Pfaff, 2002). Many of these cellular events are driven by receptor-mediated cell signalling, interpreting signals for example from shh, Wnts and BMPs (Cayuso and Marti, 2005; Ulloa and Briscoe, 2007). These overlapping signalling networks are modulated in part by cross talk with pathways governed by protein tyrosine phosphorylation, in turn controlled by protein tyrosine kinases (PTKs) and protein tyrosine phosphatases (PTPs). Phosphotyrosine signals govern isthmus-induced patterning (Sato et al., 2004), oligodendrocyte production (Pringle et al., 2003), and MN production and survival (Gauthier et al., 2007; Huang and Reichardt, 2003; Jungbluth et al., 1997; Ke et al., 2007). The proliferation of neural stem cells and progenitors also requires receptor tyrosine kinase (RTK) signalling (reviewed in (Cameron et al., 1998). Although tyrosine kinase actions are generally well defined, the roles of the tyrosine phosphatases during neurogenesis are poorly understood. A family of 21 receptor-like PTPs (RPTPs) exists (Alonso et al., 2004; Stoker, 2005; Tonks, 2006) and some of these control axon growth and guidance (Burden-Gulley et al., 2002; Ensslen-Craig and Brady-Kalnay, 2004; Rashid-Doubell et al., 2002; Shintani et al., 2006) and neuronal survival (Sakaguchi et al., 2003; Tisi et al., 2000). Dynamic expression of RPTP genes has been observed in the early developing brain and spinal cord (Chilton and Stoker, 2000; Gustafson and Mason, 2000; Ivanova et al., 2004; Sommer et al., 1997), raising the possibility that RPTPs also play a part in controlling neurogenesis and initial neuronal maturation. Gene deficiency models in mice have not currently led to a greater understanding of this area. For example, concomitant loss of both the PTPσ and PTPδ genes does lead to loss of spinal motor neurons, but only long after the MN pools have developed normally and extended axons (Uetani et al., 2006).

PTPγ is an avian RPTP expressed in the early spinal cord. It is a type V RPTP, expressed in the first spinal interneurons in chick (Gustafson and Mason, 2000) and later more broadly in the spinal cord (Chilton and Stoker, 2000). The cellular role of PTPγ in the cord is unknown, but the protein has been implicated in suppressing neurite formation in PC12 cells (Shintani et al., 2001) and suppressing the growth of breast cancer cells (Liu et al., 2004). To assess the early functions of PTPγ during neurogenesis we have used gain- (GOF) and loss-of-function (LOF) approaches in the early chick spinal cord. Our results suggest that PTPγ has several interrelated functions in controlling proliferation, survival and adhesion of progenitors and precursors.

## Results

### Expression pattern of PTPγ in the early avian spinal cord

**Fig. 1:**
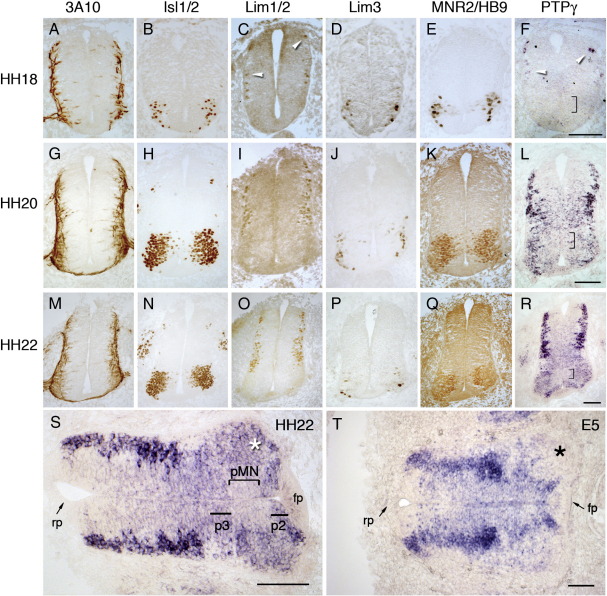
Expression of PTPγ in the early spinal cord. Expression of the chick PTPγ gene was detected with in situ hybridization at stages HH18 (F), HH20 (L), HH22 (R, S) and HH26–27 (E5) (T) and compared to localisation of neurofilament-associated protein 3A10 (A, G, and M), Lim homeodomain factors Isl1/2 (B, H, N), Lim1/2 (C, I, O), Lim3 (D, J, P) and Mnr2/HB9 (E, K, and Q). Early dorsal interneurons express both Lim1/2 and PTPγ (white arrowheads; C and F). Panel S is an enlarged, rotated version of R to compare with expression at HH26–27 (T). Square brackets demarcate the pMN domain (F, L, R, and S). In S and T: rp, roof plate; fp, floor plate; the p2 and p3 regions are demarcated in S; stars indicate motor horns Scale bar = 50 μm (A–S), 100 μm (T).

By stage HH15 of chick embryogenesis, early dorsal spinal interneurons and brachial motor neurons (MNs) of the lateral motor column (LMC) are being born (Holliday and Hamburger, 1977). Over 95% of LMC MNs are produced by stage HH24, followed by motor pool remodelling (Hollyday, 1980; Landmesser, 1978). PTPγ had been previously observed in the early spinal cord (Chilton and Stoker, 2000; Gustafson and Mason, 2000), but its precise temporospatial expression pattern was not defined. PTPγ mRNA expression was thus analysed in more detail at HH18, HH20 and HH22. At HH18, PTPγ mRNA colocalised only in dorsal, Lim1-expressing interneurons, as previously described (Gustafson and Mason, 2000) (Fig. 1C and F). At HH20, expression is maintained dorsolaterally, but also spreads extensively ventrally (Fig. 1L). Expression is most prominent in lateral regions in maturing motor neurons and interneurons. Lesser expression is found throughout the mediolateral axis, including most ventricular progenitor cells. Expression at HH22 is similar (Fig. 1R and S). Expression is present in the motor neuron progenitor domain (pMN) at HH20-22, but is relatively lower than in the progenitor domains immediately above (P2) and below (P3) (Fig. 1L, R, and S; Supp. Fig. 2). At E5 (HH26-27), there is much reduced expression in the motor horns (star, Fig. 1T), while dorsal interneurons and ventricular cells maintain expression.

### PTPγ loss-of-function (LOF) and neurogenesis

**Supplementary Fig. 1:**
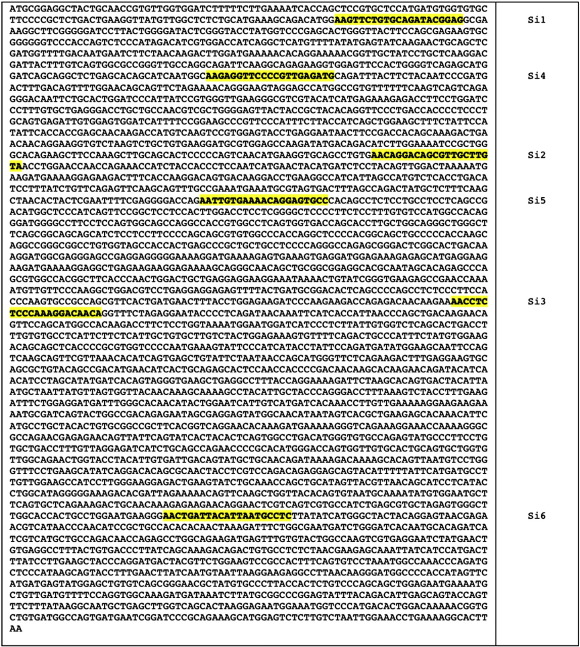
The complete cDNA sequence of the full-length form of chick PTPγ is shown. The six shRNA target sequences Si1–Si6 are shown in yellow text boxes, with their respective names on the right.

**Fig. 2:**
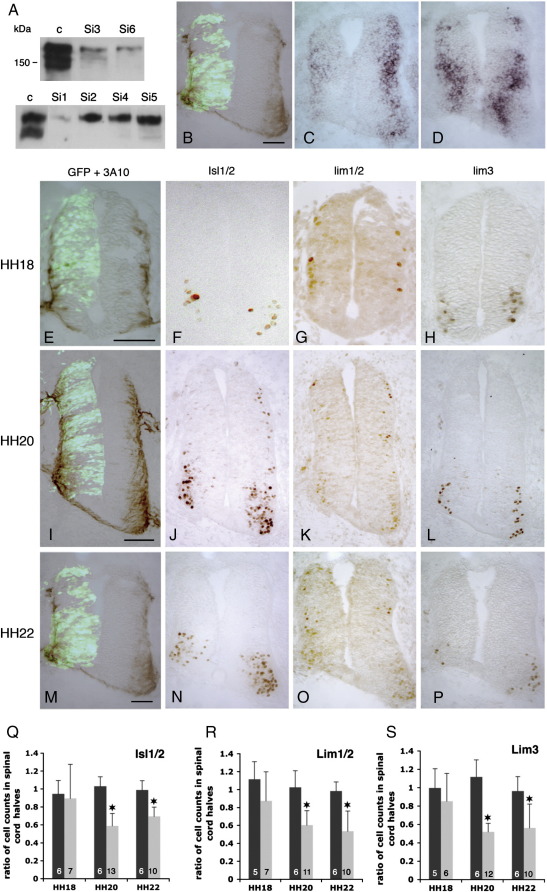
PTPγ-directed shRNA knock-down. A, immunoblots showing PTPγ protein in transfected 293T cells. PTPγ expression vector was co-transfected with either control shRNA Silencer vector (lanes c), or shRNA vectors Si1–6. Densitometry data are in Suppl. Fig. 9. Panels B–D show a HH22 neural tube electroporated with Si3 and GFP vector, after immunolabelling with 3A10 (B), or in situ hybridisation with either PTPγ (C) or NeuroM (D) riboprobes. E–P show loss of neurons after Si-3 treatment. Embryos were electroporated with Si3 at HH10-11 and fixed at HH18 (E–H), HH20 (I–L) and HH22 (M–P; the same embryo as in 2B–D). Brachial sections are shown, immunostained for Isl1/2 (F, J, N), Lim1/2 (G, K, O) and Lim3 (H, L, and P). Panels E, I and M show 3A10 staining and co-electroporated GFP; control embryos are in Suppl. Fig. 4. Scale bars = 50 μm. Ratios of labelled cell numbers on electroporated versus non-electroporated spinal cord sides are shown in Q–S. Black columns represent negative control shRNA treatments, grey columns represent Si3 treatments. Numbers within bars show the number of embryos used. Asterisks indicate statistical significance (*P* < 0.01; Student *T*-test).

Short hairpin RNA (shRNA)- and siRNA-mediated suppression of gene function is an effective technique in ovo (Harris et al., 2008; Katahira and Nakamura, 2003; Pekarik et al., 2003; Wakamatsu et al., 2007). RNA interference was used here to induce PTPγ LOF. Six shRNAs against PTPγ were cloned into pSilencer 1.0-U6 (Si1-Si6; Supp. Fig. 1). The sequences of these short hairpins had homology only to PTPγ when searched with BLASTN, thus they should be specific to PTPγ and no other PTP in the avian genome. To control for non-specific electroporation or shRNA off-target effects, a GFP expression vector and a negative control pSilencer 1.0-U6 vector (Ambion; see Experimental methods) were used. Vectors were co-transfected with a full-length, wild type PTPγ expression plasmid in 293T cells, where the most effective ones, Si1, Si3 and Si6, consistently induced 80–90% knockdown of PTPγ (Fig. 2A; quantified in Suppl. Fig. 3). As current anti-PTPγ antibodies do not work in immunohistochemistry, we used in situ hybridisation to visualise the Si vector-induced suppression of PTPγ mRNA in tissues (Fig. 2C). NeuroM, which is expressed in a pattern that partially overlaps with PTPγ, showed no reproducible alteration (Fig. 2D).

Vectors Si1, Si3 and Si6 were electroporated separately into HH10-11 neural tubes in ovo and examined at HH18, HH20 and HH22. Similar electroporations were performed with the control Silencer vector. This developmental window includes (i) early neuron production when there is little or no PTPγ expression (Fig. 1F), (ii) the onset of widespread PTPγ expression (HH20) and (iii) a point near the end of brachial MN production (HH22). In each electroporated spinal cord, neuronal numbers were counted on both the electroporated and contralateral sides, and a ratio of these was generated. The means of these ratios are graphed in Fig. 2Q–S, where comparison can be made to the negative control embryos. At HH18, electroporated cords had normal symmetry, neuronal patterns and numbers (Fig. 2E–H and Q–S). At HH20 and HH22, many Si3-electroporated spinal cords exhibited morphological distortion, predominantly with atrophied ventral horns (Fig. 2I–P). Immunostaining for Isl1/2, Lim1/2 and Lim3 confirmed that significant neuronal precursor losses began between HH18 and HH20, during the onset of widespread PTPγ expression (Fig. 2Q–S). In contrast, control shRNA-electroporated spinal cords were predominantly normal, with only occasional, minor tissue distortion and no significant change in precursor numbers (Fig. 2Q–S; Supp. Fig. 4).

**Fig. 3:**
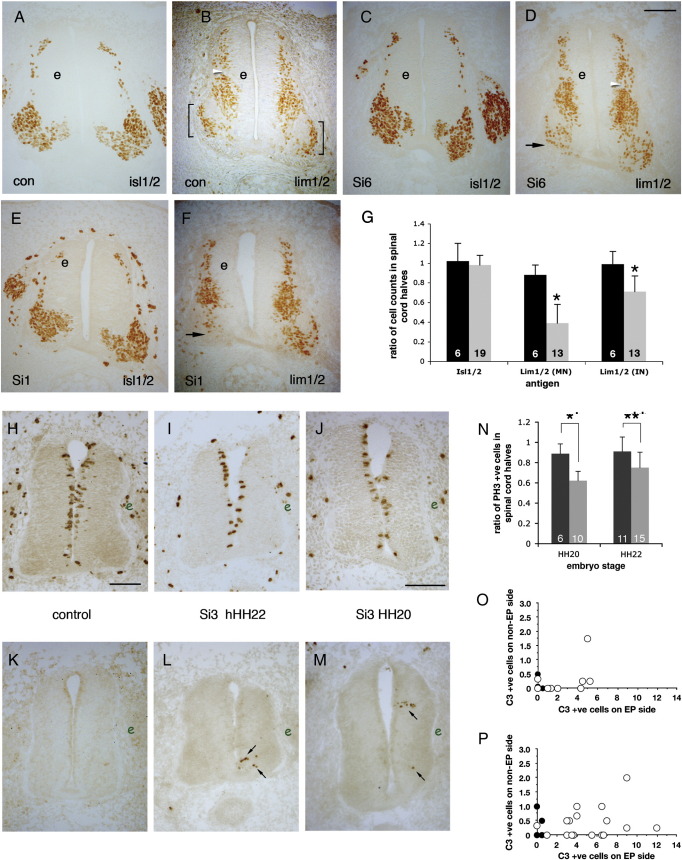
PTPγ loss-of-function effects. Panels A–G demonstrate permanent loss of Lim1/2-expressing neurons. Embryos were electroporated at HH10–11 with either a GFP vector (A and B) or shRNA vectors (C–F) and developed to HH26–27 (E5). Brachial sections were immunostained for Isl1/2 (A, C, and E) and Lim1/2 (B, D, and F). Square brackets indicate the LMCl population of Lim1-expressing motor neurons (B). Treatment with either Si1 (E and F) or Si6 (C and D) results in relatively normal Isl1/2 expression, but reduced Lim1/2-expression in LMCl (arrows in D and F); “e” indicates electroporated side. Isl1/2- and Lim1/2-expressing cells were counted on both sides of each spinal cord and ratios generated (G). Means and standard deviations of these ratios are shown for embryos treated with control shRNA (dark bars), Si3 (grey bars for Isl1/2), or a combination of Si1, Si3 or Si6-treated embryos (grey bars, Lim1/2). The Lim1/2(MN) bars represent motor neuron counts in ventral horns only; Lim1/2(IN) bars represent dorsal interneuron counts (dorsal to white arrowheads in B and D). The numbers within columns show sample sizes. Asterisks indicate statistical significance (*P* < 0.01; Student *T*-test). Panels H–P demonstrate mitotic reduction and increased apoptosis after PTPγ shRNA treatment. Embryos electroporated with Si3 (I, J, L, and M) or negative control shRNA vector (H and K) (electroporated side marked e) were fixed at HH20 and HH22 and immunostained for phosphohistone-3 (H–J) or activated caspase-3 (K–M). Phosphohistone-positive cells were counted and graphed (N). Negative controls, black columns; PTPγ shRNA Si3, grey columns. Numbers in columns indicate sample size. Asterisks represent statistical significance (** *P* < 10^− 5^; * *P* < 0.001). O and P show counts of activated caspase-immunostained cells (HH20, O; HH22, P); each spot represents one embryo, with open circles representing Si3 electroporated embryos and closed circles representing control shRNA-treated embryos. Arrows in L and M indicate caspase-expressing cells. Scale bars = 100 μm (A–F), 50 μm (H–M).

HH18–HH22 coincides with the relatively late birth period of Lim1-expressing LMCl neurons in the brachial region (Holliday and Hamburger, 1977; Sockanathan and Jessell, 1998; Whitelaw and Hollyday, 1983). LMCl neuron precursors initially express Isl-1 (prior to HH22) and then briefly coexpress Lim-1 before extinguishing Isl-1 after cessation of mitosis (Tsuchida et al., 1994). At HH26–27 (E5), spinal cords electroporated with Si1, Si3 or Si6, still showed reduced motor horn size (38/87 visibly affected) (Fig. 3A–F) and the total numbers of LMC neurons, labelled with Isl-1/2, were not significantly altered, although there was histological perturbation of the motor pool structure (see Cell adhesion and migration). Significantly, however, there was a deficiency in Lim1/2-expressing neurons of the LMCl and to a lesser degree the dorsal interneurons (Fig. 3G) (Tsuchida et al., 1994). The less effective Si vectors Si4 and Si5 (*n* = 22, data not shown), and the control shRNA (Fig. 3G), did not significantly affect the LMCl population. From the collective Si1, Si3 and Si6 data, we also believe that the effectiveness of the shRNA begins to wear off by HH22, due to plasmid dilution. For example, the numbers of Isl1/2 and lim3 positive precursors slightly recover by HH22 compared to HH20 (Fig. 2) and the same holds for phosphohistone labelling (Supp. Fig. 4). A similar effect is seen with MNR2/HB9 (data not shown).

Our data from HH18 to H27 thus indicate that PTPγ is a necessary factor for optimal generation or survival of spinal neurons, with MNs generated in the HH18–HH22 window being particularly sensitive under the technical approach used.

### Reduction in mitoses

To investigate how spinal cord atrophy might be occurring, electroporated cords were screened for phosphohistone-3 levels. Control shRNA induced a small reduction in mitoses as indicated by phosphohistone-positive cells (Fig. 3N), but Si3-electroporated embryos had 40% fewer mitotic cells at HH20 (*P* < 10^− 3^ compared to control) and 22% fewer at HH22 (*P* < 10^− 5^) (Fig. 3N), indicating that PTPγ loss-of-function suppresses the mitotic rate in progenitors.

**Fig. 4:**
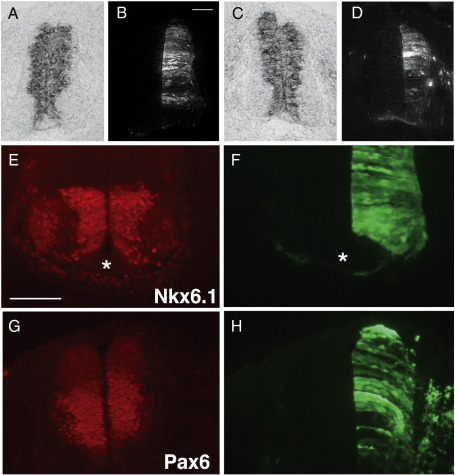
Notch signalling and homeodomain factors. Embryos were electroporated with control shRNA vector (A and B) or Si3 (C–H) at HH11 and fixed at HH22. Sections were hybridised with Hes5-1 probe (A and C), or immunostained for Nkx6.1 (E) and Pax6 (G). Green channels (B, D, F, and H) show co-expressed GFP. Si3 does not alter the general pattern or level of Hes5-1 or the homeodomain proteins. Asterisks in E and F show floor plates. Scale bar = 50 μm.

We addressed whether PTPγ action was likely to be upstream or downstream of Notch signalling, a primary regulator of proliferation versus differentiation in neural progenitors (Yang et al., 2006). Hes5-1 expression, a primary readout of Notch (Fior and Henrique, 2005), remains normal in Si3-treated tissues (Fig. 4A–D). Moreover, homeodomain proteins whose expression is maintained by Notch 1 signalling, such as Pax6 and Nkx6.1 (Yang et al., 2006), are also normally expressed (Fig. 4E and G). The effects of PTPγ signalling thus appear to be either downstream or independent of Notch.

### Increased apoptosis

Very low levels of cell death normally occur in the spinal cord at stages HH20 and HH22 (Homma et al., 1994) and this is prior to the main onset of caspase-dependent programmed cell death of brachial MNs at HH22 (Li et al., 1998). Control shRNA did not change the rate of apoptosis (Fig. 3H, K, O, and P). In contrast, activated caspase 3 expression was significantly elevated after Si3 treatment (9.7 fold increase at HH20 [*p* < 10^− 4^]; 8.9 fold increase at HH22 [*p* = 0.005]) (Fig. 3I, L, M, O, and P). The increased apoptosis accompanying PTPγ loss-of-function implicates this enzyme in sustaining the survival of progenitors and precursors in the early spinal cord.

### Elevated PTPγ suppresses neurogenesis

**Fig. 5:**
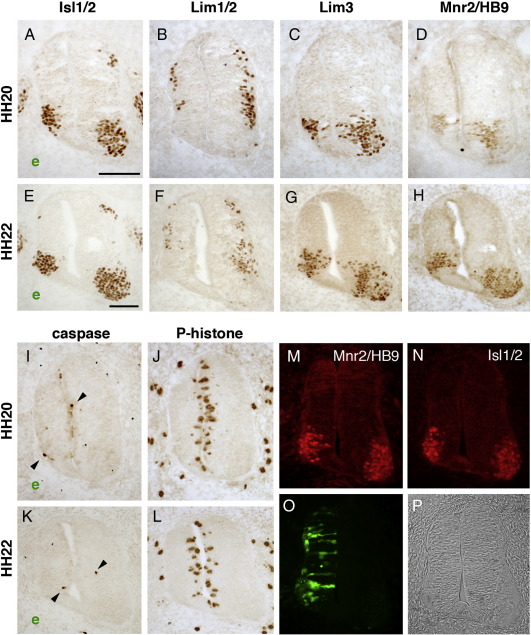
PTPγ gain-of-function. Electroporation of wtPTPγ vector at HH10–11 (e, electroporated sides), causes losses of ventral motor neurons and dorsal interneurons, observed at HH20 (A–D, I, and J) and HH22 (E–H, K, and L). Sections were immunostained for Isl1/2 (A and E), Lim1/2 (B and F), Lim3 (C and G) and MNR2/HB9 (D and H). Immunostaining for activated caspase-3 (I and K) shows few apoptotic cells (arrowheads). Detection of phosphohistone-3 (J and L) reveals reduced mitoses after wtPTPγ expression. The counts of neurons are graphed in Supp. Fig. 4. M–P show a HH20 spinal cord electroporated with pTMintγ vector, showing expression of Mnr2/HB9 (M) and Isl1/2 (N). Scale bar = 50 μm.

To address further the role of PTPγ, gain-of-function (GOF) experiments were carried out using a wild type PTPγ (wtPTPγ) expression vector. Increased PTPγ expression caused prominent loss of ventral tissue (Fig. 5A–H) whereas control electroporation did not (Supp. Fig. 4D–G). Unlike with LOF treatment, however, the dorsal tissue was more clearly and frequently atrophied after PTPγ GOF. Underlying these histological changes, MNs and dorsal interneurons were both significantly reduced in numbers (Fig. 5; Supp. Fig. 5). PTPγ overexpression did not alter Pax3 or Nkx6.1 expression (data not shown). The underlying basis for neuronal loss appears to be at least in part a reduction in mitosis again, as judged with phosphohistone 3 (Fig. 5J and L; Supp. Figs. 4 and 5). However, GOF did not activate caspase, again in contrast to LOF (Fig. 5I and K). This suppression of proliferation is likely to be cell-autonomous, since expression of a form of PTPγ lacking most of its extracellular domain, gave similar phenotypes (Fig. 5M–P).

### PTPγ modulates Wnt/β-catenin signalling

A possible target of PTPγ could be the canonical Wnt/β-catenin signalling pathway, since this is a major regulator of proliferation and differentiation in the cord, activated by Wnt signals in a high to low dorsoventral gradient (Megason and McMahon, 2002). β-catenin modulates cadherin-dependent cell–cell adhesion at adherens junctions of epithelia and also acts as a direct transcriptional regulator in complexes with TCF/LEF proteins (Clevers, 2006; Lilien and Balsamo, 2005). These nuclear and junctional activities of β-catenin can both be regulated by its tyrosine phosphorylation and several phosphatases have been implicated in this process (Lilien and Balsamo, 2005; Sallee et al., 2006).

**Fig. 6:**
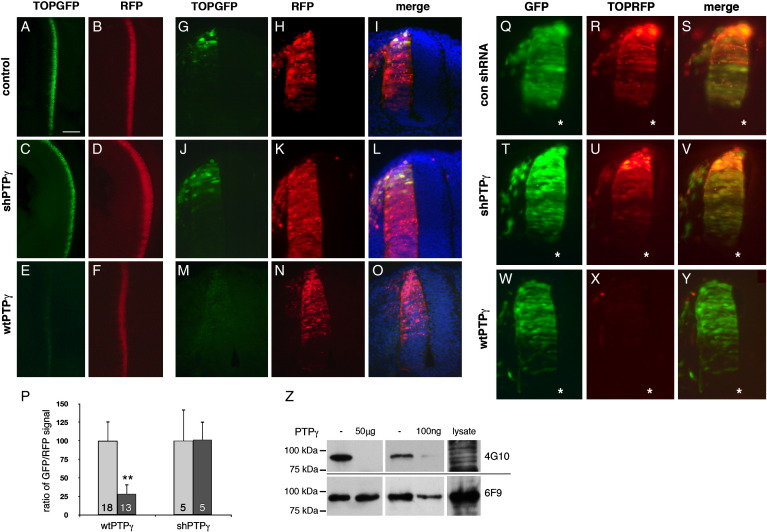
PTPγ gain-of-function suppresses TCF-dependent transcription. To detect TCF activity, embryos were electroporated with TOPGFP along with an electroporation reporter (RFP) and either a control shRNA plasmid (A, B, and G–I), the Si3 vector (shPTPγ; C, D, and J–L) or wtPTPγ expression vector (E, F, and M–O). A–F, wholemount images of embryo trunks viewed dorsally. G–O are sections of similar embryos (dorsal top), showing GFP expression, RFP expression and image merges with DAPI. A sharp, dorsoventral gradient of TOPGFP is visible (G and J). Total GFP and RFP signals were quantified and GFP/RFP ratios determined and graphed (P) (see Experimental methods). In P, each column contains the sample size; error bars represent SD (* *P* < 0.01; ** *P* < 0.001). Q–Y show similar assays with a more sensitive TOPRFP reporter (and GFP electroporation reporter), in HH22 spinal cords. TOPRFP reveals a full dorsoventral gradient of TCF activity, which is similar in control shRNA- and Si3-treated embryos (R and U, respectively). X shows almost complete extinction of TOPRFP signal after wtPTPγ expression. Z shows in vitro dephosphorylation of β-catenin protein by PTPγ. β-catenin was immunopurified from 293 T cells and incubated with either 50 μg or 100 ng purified human PTPγ D1/D2 catalytic domains. Samples were immunoblotted to detect phosphotyrosine and β-catenin. Whole cell lysate samples are also shown. Scale bar = 500 μm (A–F) 50 μm (G–Y).

Blockade of TCF/LEF signalling function in the chick spinal cord gives similar gross phenotypes to those seen after PTPγ perturbation (Megason and McMahon, 2002). To examine if PTPγ influences Wnt/β-catenin signalling in ovo, we assessed TCF activity with a pTOPGFP reporter. With this vector, GFP expression reflects the binding of an activated β-catenin-TCF/Lef complex to 4 consensus TCF/Lef binding sites in the GFP promoter (Dorsky et al., 2002). pTOPGFP was introduced at HH11 along with either Si3, control shRNA vector, wtPTPγ vector, or a negative control plasmid. As expected, control embryos at HH20 showed a steep dorsoventral gradient of TOPGFP (Fig. 6G) (Megason and McMahon, 2002). As judged by GFP/RFP ratios, Si3 did not significantly affect GFP expression (Fig. 6C, D, J–L, and P). In contrast, wtPTPγ strongly suppressed TOPGFP expression by at least 80% (Fig. 6E, F, M–O, and P). As this pTOPGFP vector was found to generate a very steep gradient and no GFP in the ventral cord, an alternative pTOPRFP vector was found that could be used to assess the ventral TCF activity level. Once again, widespread expression of wtPTPγ dramatically suppressed TOP-RFP expression and this was observable across the entire spinal cord (Fig. 6W–Y). Si3 expression had no obvious effects (Fig. 6T–V).

**Fig. 7:**
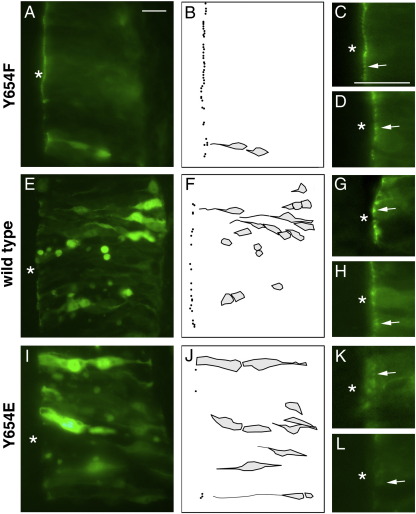
Localising Y654 variants of β-catenin. Embryos were electroporated with GFP-β-catenin expression vectors at HH11, fixed at HH20 and sectioned. Representative images show localisation of GFP-β-catenin fusions proteins with Y654F β-catenin (A–D) wild type β-catenin (E–H) and Y654E β-catenin (I–L). C, D, G, H, K and L are enlargements of ventricular surfaces from further embryos, with arrows indicating adherens junctions; junctions are sharp and defined in C, D, G, and H, but faint and diffuse in K and L. Panels B, F and J are schematic tracings of the panels to their left, showing both adherens junctions (black dots) and cell bodies that contain cytoplasmic GFP-catenin at levels significantly above background (some cells are above or below the main focal plane). Asterisks indicate spinal cord lumens. Scale bars = 50 μm.

In the Wnt pathway, β-catenin is therefore a potential target for wtPTPγ. Tyrosine phosphorylation of β-catenin alters its ability to localise to cadherins and can also influence its transcriptional potential (Lilien and Balsamo, 2005; Sallee et al., 2006; Yan et al., 2006). We initially attempted to examine endogenous levels of β-catenin phosphorylation in HH22 spinal cords. However, the endogenous phosphorylation level was very low and we could not reliably determine whether PTPγ was altering this pattern. Similarly, antibodies to phosphoY654 and phosphoY489 (Rhee et al., 2007), were not sensitive enough to detect phospho-catenin in ovo. Instead, therefore, we tested whether the phosphorylation state of β-catenin at one of its regulatory tyrosines, Y654, influences β-catenin compartmentalisation between its active sites, the nucleus and adherens junctions (AJ), in spinal cord cells. Compared to cells expressing wild type β-catenin-GFP, we found that β-catenin-GFP fusion proteins with Y654-F mutations (mimicking a dephosphorylated state) (Murase et al., 2002), localised predominantly to AJ (Fig. 7A–D). In contrast, Y654-E mutations (mimicking phosphorylation) caused the protein to localise in the cytoplasm and nucleus predominantly (Fig. 7GI–L). Tyrosine phosphorylation of β-catenin, at least at Y654, is thus capable of altering protein localisation and thus potentially β-catenin function in the cord. Interestingly, tyrosine phosphorylation of β-catenin may be in itself insufficient to alter signalling in these cells, since the Y654E mutation might be expected to have a dominant-active phenotype, inducing tissue hypertrophy, but it does not (data not shown). In a second approach, we tested whether PTPγ could dephosphorylate β-catenin in vitro. Tyrosine-phosphorylated β-catenin was immunopurified and treated in vitro with purified catalytic domains of human PTPγ (Barr et al., 2009b). WtPTPγ did indeed efficiently dephosphorylate β-catenin (Fig. 6Z). Thus, among the potential targets of PTPγ in ovo, β-catenin remains one candidate.

### Cell adhesion and migration

**Fig. 8:**
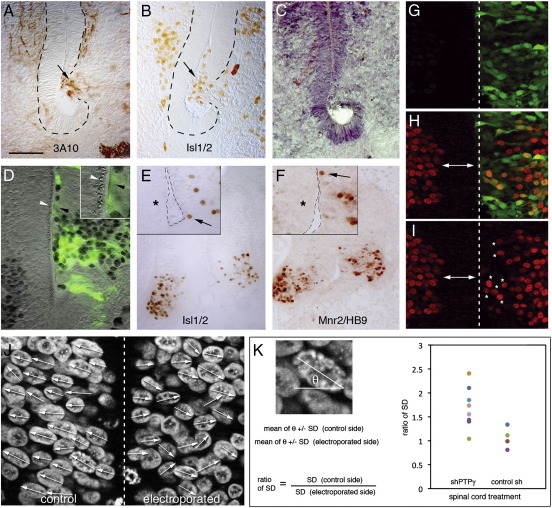
Mediolateral mislocation of neurons and polarity defects. Embryos electroporated with Si3 at HH10–11 (electroporated on the right), were fixed at either HH31 (E7) (A–C; same embryo), HH26–27 (E5) (D) or HH22 (E, F; same embryo) and stained with H&E (C) or immunostained with 3A10 (A), Isl1/2 (B, D, E; D has combined DIC/fluorescence optics) or MNR2/HB9 (F). Panels G–I show an HH22, Si3-electroporated embryo (midline in the centre) with Isl1/2 immunofluorescence (H and I, red cells; Zeiss Apotome micrographs) and GFP showing the extent of electroporated tissue (G). Medially misplaced, heterotopic neurons in ventricular zones are indicated by arrows in A and B (the limits of the disrupted ependymal tissue are shown with dashed lines), arrowheads in E and F and asterisks in I. In D, the ventricular, progenitor tissue (granular appearance; white arrowhead, inset) is replaced by electroporated, neuronal tissue (black arrowhead, inset). Insets in E and F show lumens outlined in dashed lines and normal progenitor zones with asterisks. Arrowheaded bars in H and I show the width of the normal progenitor tissue, devoid of Isl1/2-expressing cells; the contralateral, electroporated region is filled with Isl1/2-expressing cells (asterisks). Scale bar = 50 μm (A–D, G–I), 100 μm (E. F), 15 μm (J). Panel J shows DAPI staining of HH22 spinal cord tissue, just dorsal to the pMN, with the midline (dashed line) demarcating electroporated (right) and control (left) sides. Arrows indicate long axis polarity of each nucleus. K, the angles of axes were measured and the standard deviations of mean angles calculated. The [control side/electroporated side] ratios of these deviations were graphed for embryos treated with either control shRNA or Si3. The greater values seen with Si3 are a reflection of more misoriented cell bodies. (For interpretation of the references to color in this figure legend, the reader is referred to the web version of this article.)

In Si-treated embryos, the pMN region was commonly atrophied. In a third of HH26–27 (E5) and HH31 (E7) embryos treated with Si3, there were also striking abnormalities in mediolateral positioning of MN precursors. These abnormal cells became embedded in the ventricular zone, ingressing into the lumen in more extreme cases (Fig. 8A–D). Concomitantly, the ventricular tissue was much reduced or was missing (Fig. 8C, black arrow; D, black arrowhead). Although this was phenotypically similar to the loss of spinal ventricular tissue and the ventricular location of Isl1-expressing neurons in Notch1-deficient mice (Yang et al., 2006), we have already noted above that Notch signals appear to be unaltered in the Si3-treated chick embryos.

The medial mislocation of neurons occurred only in the pMN, not dorsally or ventrally. It was clearly visible by HH22 and thus must be initiated earlier. Approximately one third of HH22 Si3-treated embryos had misplaced neurons (Fig. 8E, F, G–I; asterisks in 8I), with ventricular and subventricular tissue already reduced in volume (asterisks in Fig. 8E and F, arrowheads in 8 H; Suppl. Fig. 6). Neuronal mislocalisation was also seen with Si1 (Suppl. Fig. 7), but not with the pSilencer negative control (Supp. Fig. 8), or another negative control shRNA vector pRFPRNAi-LacZ (Ark genomics; data not shown). Optical sectioning also showed that most heterotopic neurons were GFP-expressing (48/49 misplaced neurons counted; Fig. 8G–I), indicating a cell-autonomous defect. There was no alteration in mitotic spindle angle distributions (Suppl. Fig. 6) (Zhong and Chia, 2008), suggesting that the balance of self-renewal and neurogenic fate was unaltered. Loss of progenitors through death, or their displacement by motor neuron precursors that fail to migrate, may instead be the most important contributing factors.

In the neuroepithelium outside the pMN, PTPγ LOF also appears to perturb cell–cell adhesion. Nuclei of the neuroepithelium are normally elongated and well aligned mediolaterally, reflecting the overall shape and polarity of the cells (Fig. 8J, left of midline). The average angles of nuclei were measured in spinal cords treated with control shRNA or Si3. The spread of angles, represented as a standard deviation from a mean angle, reflects the degree of relative alignment across the population. PTPγ LOF increased the spread of angles significantly and also increased the number of unpolarised nuclei (Fig. 8J and K), both a reflection of more random orientation. PTPγ is therefore required for movement of motor neuron precursors and, more broadly, the orderly cell–cell adhesion in the neuroepithelium.

## Discussion

Tight control of the proliferation of neural progenitors allows for the generation of suitable numbers and patterns of neurons in the spinal cord. RPTPs such as PTPγ can now be added to the list of enzymes that play a part in controlling these events. After the onset of widespread expression of PTPγ between HH18 and HH20, this phosphatase plays several, potentially interrelated roles. When PTPγ expression is perturbed either up or down, the proliferation of neural progenitors reduces and, in the case of PTPγ LOF, cell death rates increase. At a molecular level one function of PTPγ may be to modulate Wnt/ β-catenin signalling in the spinal cord, possibly through phosphorylation of β-catenin or some other pathway target. The phosphatase also plays roles in maintaining neuroepithelial cell adhesion and polarity, as well as potentially facilitating the lateral migration of motor neuron precursors.

### PTPγ and spinal neurogenesis

While some studies have shown that RPTPs such as PTPσ, PTPδ and PTPRO control motor axon behaviour or the later survival of MNs, gene-deficient animals have not shed much light on earlier roles for RPTPs in the spinal cord (Lamprianou et al., 2006; Uetani et al., 2006). The early avian spinal cord expresses several RPTP genes (Chilton and Stoker, 2000; Gustafson and Mason, 2000) and we now show that LOF and GOF in PTPγ generates defects in neurogenesis. This is intriguing given that PTPγ also acts as a growth suppressor in breast cancer cells (Liu et al., 2004) and can suppress differentiation in PC12 cells (Shintani et al., 2001). Although neurogenesis was normal in shRNA-treated LOF embryos prior to HH18, decreased neurogenesis and precursor numbers occurred thereafter, coincident with the onset of widespread PTPγ expression in progenitor and precursor cells. The ultimate neuronal deficits by E5 were largely reflected in a permanent loss of Lim1/2-expressing interneurons in the LMC. Although total neuron numbers, judged by Isl1/2 were little altered on average, many of these would have been born prior to HH18 (Holliday and Hamburger, 1977; Whitelaw and Hollyday, 1983). Moreover, the histological arrangement of the Isl1/2-positive neurons was clearly disturbed in many instances. We believe that the period of shRNA treatment between HH18-HH22 has targeted those neurons whose birth temporally coincides with that window, in particular with the LMCl population (Holliday and Hamburger, 1977; Whitelaw and Hollyday, 1983). Since we believe that shRNA effectiveness declines after HH22, it is likely thereafter that some compensatory recovery of non-lim1/2 neuron numbers occurs, although this remains to be demonstrated directly. The study therefore supports a critical requirement for PTPγ at least during the HH18–HH22 window of neurogenesis.

Although perturbation of PTPγ expression either up or down can suppress mitoses, this appears to occur through different mechanisms. With PTPγ GOF, suppression is most likely due to loss of TCF activity. TCF-driven transcription is critical for maintenance of spinal proliferation, downstream of Wnt/β-catenin signals (Megason and McMahon, 2002). PTPγ LOF in contrast does not alter TCF signalling, but instead induces distinct phenotypes. First, there is an increase in cell death in the progenitor and mantle zones. Second, we see a loss of mediolateral alignment of neuroepithelial cells and defects in lateral migration of motor neuron precursors. The latter observation indicates that PTPγ may be necessary for maintaining appropriate cell–cell adhesion in many cells after HH18. The increased apoptosis could be a consequence of this aberrant cell–cell adhesion and associated cell signalling, although a direct influence of PTPγ over cell survival is also possible. The experiments also showed that Notch signalling was not perturbed and neither was the pattern of mitotic spindle angles, although a straightforward relationship between spindle angle and cell fate in the spinal cord has recently been called into question (Wilcock et al., 2007). These data indicate that PTPγ may not be primarily affecting the balance of proliferation versus differentiation. Therefore, the decreased neurogenesis following PTPγ LOF most likely results from a perturbation of cell adhesion and progenitor tissue structure, and increased apoptosis; these could also be directly interrelated.

### Defects in motor neuron development

**Supplementary Fig. 2:**
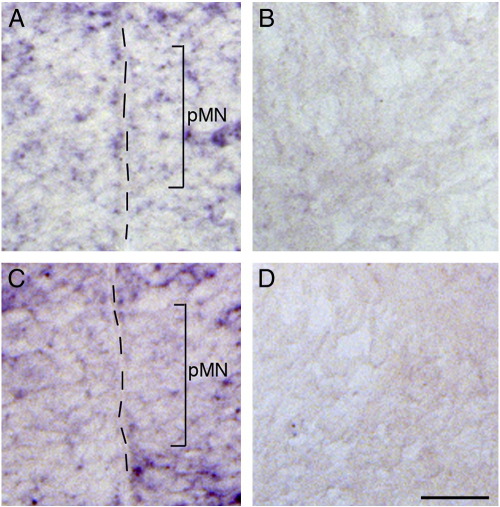
PTPγ expression in the pMN domain. Enlarged images are shown of the motor neuron progenitor region (pMN) of HH20 (A, B) and HH22 (C, D) embryos shown in Fig. 1L and R, respectively. Left hand panels (A and C) show weak but visible in situ signal for PTPγ mRNA in the ventricular region of the pMN (square brackets) either side of the midline (dashed line), compared to the right hand panels (B and D) that show neighbouring connective tissue, which is devoid of signal.

**Supplementary Fig. 3:**
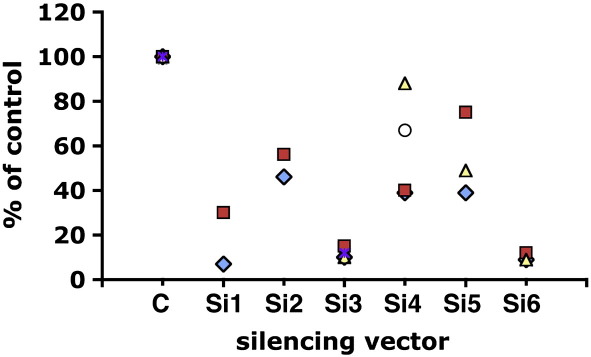
Quantitation of decreased PTPγ protein band intensities after shRNA treatment of 293T cells. Protein bands were scanned and normalised for luciferase content of respective lysates and controls were set at 100%. Each coloured symbol represents a separate experiment (*n* = 5, c; *n* = 2, Si1; *n* = 2, Si2; *n* = 5, Si3; *n* = 4, Si4; *n* = 3, Si5; *n* = 3, Si6).

**Supplementary Fig. 4:**
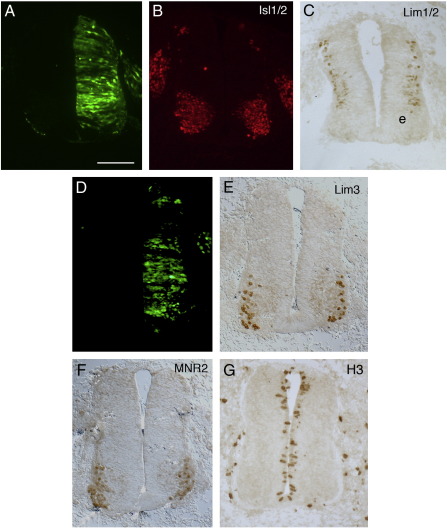
Control HH22 embryos with normal phenotypes, electroporated with GFP plus either the random shRNA silencer vector (A–C), or the control alpha18 plasmid (D–G). A and B show an embryo with GFP expression (A) and Isl1/2 immunolabelled in the same section with Cy3 (B). Panel C shows a different embryo labelled immunohistochemcially for Lim1/2 (e, electroporated side). D–G show one embryo with GFP (D) and neighbouring sections immunolabelled for Lim3, MNR2 and phosphohistone 3 (H3). Scale bar = 100 μm.

**Supplementary Fig. 5:**
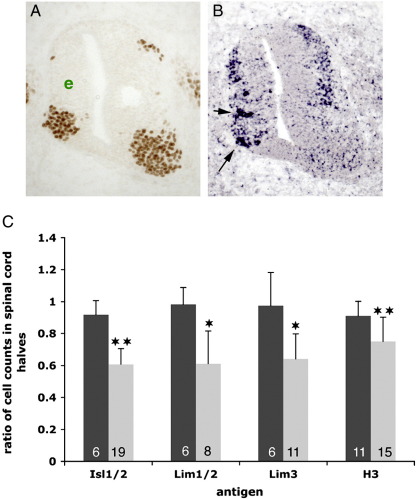
Gain-of-function for PTPγ. Panels A and B show an HH22 embryo elctroporated at HH11 with wtPTPγ expression vector, stained for Isl1/2 (A; the same embryo as in Figure 5E) or by in situ hybridisation for PTPγ mRNA (B). Panel B shows the increased PTPγ mRNA expression on the electroporated side (marked e in panel A). C, quantitation of neuron numbers after wtPTPγ expression in ovo (see Fig. 5). Embryos were electroporated with control pIRESGFP vector (black bars) or wtPTPγ vector (grey bars) and sections immunostained. Cells immunopositive for Isl1/2, Lim1/2, Lim3, or phosphohistone 3 (H3) were counted on both sides of each cord. Ratios were generated and means are graphed, with standard deviations shown (asterisks show statistical significance with Student *T*-test; * *P* < 0.008; ** *P* < 10^− 4^). Numbers in columns indicated embryo sample sizes.

**Supplementary Fig. 6:**
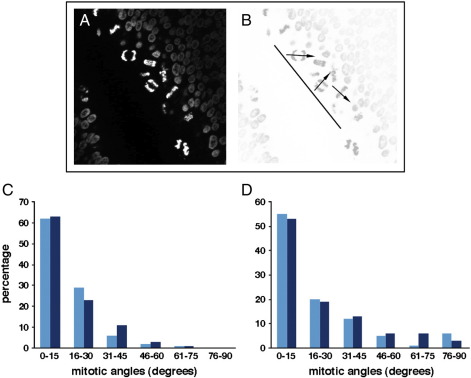
Mitotic angles are unchanged after PTPγ perturbation. Spinal cords were electroporated at HH11 with expression vectors and fixed at HH22 before sectioning and staining with DAPI (A; put AB in figure). Angles of anaphase nuclei were measured (B) with respect to the inner face of the ventricles; 0° represents horizontal cleavages (example 1), 90° represents orthogonal cleavages (example 2). C, graph of angles, binned into 15° groups. Light bars are embryos expressing control shRNA, dark bars expressing shPTPγ Si3. D, graph of angles in embryos expressing negative control vector (light bars) or wtPTPγ expression vector (light bars). At least 100 nuclei were measured from a minimum of 5 different embryos in each data set. No significant differences were observed between controls and experimental samples.

**Supplementary Fig. 7:**
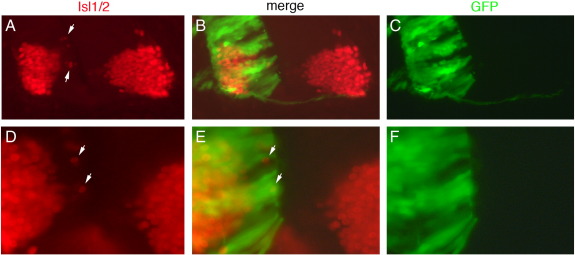
Motor neuron heterotopia after treatment with shPTPγ vector Si1. Si1 DNA was electroporated at HH10–11, and HH22 sections were analysed with immunofluorescence microscopy. An example is shown of Isl1/2-expressing motor neurons aberrantly localised to ventricular sites (arrows). A and D are two sections from one embryo, with D at enlarged scale. Scale bar = 50 μm (A–C), 25 μm (D–F).

**Supplementary Fig. 8:**
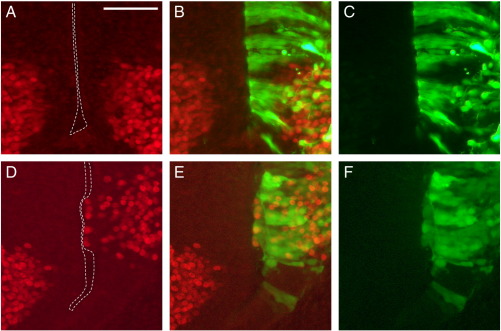
Comparison of pMN domains after electroporation of negative control shRNA vector versus Si3. The control embryo shown (A–C) was electroporated along with the GFP tracker vector at HH10–11 and sectioned at HH22 before immunostaining for Isl1/2 (A–C). The ventricular zone is intact and devoid of motor neuron precursors. The Si3-expressing spinal cord shown (D–F), has a severe defect with motor neuron precursors embedded in the ventricular layer and progenitor tissue missing. The ventricular surfaces are indicated by dashed white lines (A and D). Scale bar = 50 μm.

The most striking effect of PTPγ LOF was the specific loss of pMN progenitor tissues in some embryos and concomitant mis-localisation of maturing MNs. This was not seen in other ventricular regions. It has been suggested that maturing MN precursors may provide feedback to progenitors, biasing them away from a MN fate (Pfaff et al., 1996). Isl-1 deficiency leads to premature MN death, where this feedback may be lost, resulting in compensatory MN production, progenitor exhaustion and the observed progenitor depletion (Chitnis et al., 1995; Pfaff et al., 1996). Although we do observe increased apoptosis associated with PTPγ LOF, this was not restricted to the pMN. Currently therefore, it remains to be determined why the pMN region is so sensitive to PTPγ LOF. For example, cells in the pMN could depend on very specific, cadherin-based adhesive functions that are perturbed by PTPγ. Alternatively, the pMN might be particularly sensitive to gene knock-down, since expression of the gene is lower there when compared to progenitors dorsally and ventrally (Fig. 1S; Suppl. Fig. 2).

The medial mislocation of MNs is frequently observed after PTPγ LOF. This might arise simply through the physical depletion of ventricular tissue. Arguing against this are two factors. First, individual neurons were sometimes mislocated in otherwise normal-looking ventricular tissues. Second, non-electroporated neurons are rarely mislocated even when extensive progenitor tissue is missing, whereas mislocated neurons were nearly all GFP-expressing (Fig. 8). This indicates a cell-autonomous defect in MN precursor localisation. One potential role of PTPγ therefore is to regulate progenitor and precursor cell movement through cell–cell or cell–matrix interactions.

### PTPγ and cell signalling

We have shown that PTPγ has profound effects on the generation, localisation and survival of neural precursors in the spinal cord. What are its likely molecular targets? The influence over cell polarity and migration could be at several levels. For example, in many cell types, tyrosine phosphorylation of cadherins and catenins control cell–cell adhesion (Lilien and Balsamo, 2005; Sallee et al., 2006). The regulation of cell–matrix interactions is also heavily dependent on integrins and associated targets of tyrosine kinases such as FAK and src (Mitra and Schlaepfer, 2006). Recent studies of the zebrafish *tab* mutation of laminin 1 demonstrates that integrin signaling and FAK activation is central to the control of interkinetic nuclear movement and neurogenesis in the neural tube (Tsuda et al., 2010). β-catenin is another candidate target. PTPγ GOF (shown here) and β-catenin LOF in mice and chick (Megason and McMahon, 2002; Zechner et al., 2003), all result in loss of ventral progenitor cells, potentially pointing towards a common basis of defective β-catenin signalling. Our data certainly show that PTPγ can suppress Wnt/β-catenin signalling through TCF in the spinal cord and that PTPγ can dephosphorylate β-catenin in vitro. Dephosphorylation of β-catenin phosphopeptides has also has been demonstrated previously (Barr et al., 2009a). However, in vitro assays of PTP specificity are notoriously unreliable and several PTPs are already known to target catenins in other cell types (Lilien and Balsamo, 2005; Sallee et al., 2006). In the chick hindbrain the RPTP PTPλ interacts with β-catenin directly, and its overexpression suppresses Wnt/β-catenin signalling and cell proliferation (Badde and Schulte, 2008). Regulation of the Wnt/β-catenin signalling pathway may be one of the shared, physiological functions of PTPγ or PTPλ in ovo, but such signaling could also be subject to a complex level of PTP redundancy. Such redundancy might explain why changes in TCF activation are observed only after PTPγ GOF, not PTPγ LOF. Alternatively, changes in β-catenin phosphorylation may be necessary, but not sufficient for TCF signalling. β-catenin phosphorylation has been shown previously to be insufficient to fully activate its nuclear function (Kim and Lee, 2001). Also, in our hands the phosphomimic Y564E β-catenin did not lead to full activation of the TCF pathway and tissue hypertrophy. Components of the Wnt/TCF pathway other than β-catenin must therefore be considered as PTPγ targets. For example, the tyrosine phosphorylation state of nuclear cofactors such as steroid receptor coactivator-3 can affect transcriptional activity of p300, a cofactor in β-catenin nuclear activity (Oh et al., 2008).

Other potential targets of PTPγ are the cadherins (Lilien and Balsamo, 2005; Sallee et al., 2006). Our observations of defective cell polarity and migration in ovo are consistent with defects in cell–cell adhesion, and MN migration does rely in part on cell–cell adhesion through cadherins (Price et al., 2002). Since cadherin function is also dependent on tyrosine phosphorylation (Lilien and Balsamo, 2005; Sallee et al., 2006), this remains an area of interest for further investigation.

In conclusion, this is the first demonstration of a role for PTPγ in the embryonic nervous system. PTPγ is a regulator of the proliferation and survival of avian spinal cord progenitors and neural precursors, playing potential roles in Wnt/β-catenin signalling, cell adhesion and the migration of motor precursors. It will be interesting to understand the role of this enzyme in more mature spinal neurons as well, since expression in these can be very high. PTPγ is one of a growing number of RPTPs, including PTPσ (Kirkham et al., 2006; Meathrel et al., 2002) and PTPλ (Badde and Schulte, 2008) that have roles in CNS neurogenesis. The actions of this enzyme family are therefore likely to be significant contributors to signalling cross talk with other known regulators of CNS growth and patterning.

## Experimental methods

### Plasmids and silencing vectors

Plasmids pCAβ-GFP and pCAβ-RFP were provided by Jonathan Gilthorpe (Umeå University, Sweden) and the *Renilla* luciferase reporter vector pRL-SV40-renilla was from Promega, UK. Full length PTPγ cDNA was provided by Lu-Hai-Wang (NIMR, Mill Hill, UK) and was subcloned in-frame with 3xFLAG in p3xFLAG-CMV14 (Sigma Aldrich). This c-terminally tagged PTPγ cDNA was then subcloned into pCAβIRESGFP (gift of Jonathan Gilthorpe), for enhanced expression in ovo. The extracellular deletion of PTPγ was constructed by fusing the amino-terminal FLAG tags of p3xFLAG-CMV25 (Sigma) to amino acid 733 of chick PTPγ. Six, PTPγ-specific short hairpins were designed using Ambion algorithms. The sequences (Supp. Fig. 1) were checked using BLASTN in NCBI to avoid non-target homologies. Annealed oligonucleotides were ligated into the pSilencer1.0 U6 vector (Ambion, USA) and plasmids were named Si1–Si6. The negative control Silencer vector contained a random hairpin with no avian homologies according to BLASTN (Ambion, USA). The β-catenin-GFP fusion vectors were obtained from Addgene Inc. USA. The TOPGFP vector was given by Randall Moon (University of Washington) and TOPRFP was given by Nobue Itasaki (National Institute for Medical Research, UK). The Hes5-1 in situ probe vector was kindly given by Domingos Henrique (Fior and Henrique, 2005).

### Cell culture and luciferase assays

Human embryo kidney 293T cells were cultured in DMEM (Sigma, UK) containing 10% Foetal bovine serum (Sigma, UK). Si vectors were co-transfected with the PTPγ expression plasmid and the Luciferase reporter, using calcium phosphatase methods. After 24 h, cells were lysed on ice in 0.25% TritonX-100, 150 mM NaCl, TrisHCl pH 7.6 with protease inhibitors (Complete, Roche). Lysates were processed for immunoblotting and luciferase assays. Twenty microlitre aliquots of lysates were assayed using the Luciferase assay system (Promega, USA.) and a Berthold Technologies luminometer LUMAT LB 9507 (Bad Wildbad, Germany).

### Immunoblotting

Lysates were subjected to SDS polyacrylamide gel electrophoresis (PAGE) and transferred to PVDF membrane. After blocking in 10% milk powder/TBST (50 mM Tris pH 7.4, 150 mM NaCl, 0.2% Tween20) overnight, the filters were probed with primary and HRP-conjugated secondary antibodies diluted in 10% Milk/TBST. The bound antibodies were detected using ECL plus (Amersham, UK).

### *In ovo* electroporation

Silencing constructs (2 μg/μl) were mixed with pCAβ-GFP DNA (3 μg/μl) and fast green dye at a volume ratio of 2.5:0.3:0.2. DNA was injected into the neural tube at embryo stage Hamburger and Hamilton (HH) 10–11 (Hamburger and Hamilton, 1992), and the targeted region was electroporated at 20 V, for 5 pulses lasting 50 ms, with 950 ms intervals, using gold-plated electrodes (BTX Inc. model 508) and an ECM830 electroporator (BTX, Inc, Hawthorne, NY, USA). After further incubation, the embryos were dissected, staged (Hamburger and Hamilton, 1992), fixed in 4% paraformaldehyde/PBS and cryosectioned at 12μm thickness.

### Immunodetection

For immunohistochemistry, slides were pretreated with 1% hydrogen peroxide in PBS for 20 min, then blocked in 4% BSA (Fraction V, Sigma) in PBS for 20 min. Primary and secondary antibodies were added for 1 hour each, with intermediate wash steps. Bright field and fluorescence microscopy was carried out using a Zeiss Axiovert, and recorded with a Hamamatsu Orca-1 camera and Openlab software (PerkinElmer UK).

To detect activated caspase-3 and phosphohistone, cryosections were placed in Declere (1x; Cell Marque Corp, California, USA) for 40 min with steam, then in freshly boiled Declere for 10 mins. Slides were washed in TBST pH 7.5 (100 mM Tris, 150 mM NaCl, 0.1% Tween 20), treated with 3% H_2_O_2_ then washed again. Slides were blocked in 0.15% Glycine, 2 mg/ml BSA, 5% Goat serum in TBST for 30 min, then primary antibodies were added overnight. After washing, slides were incubated with secondary antibody, washed, then detection was performed with ABC (Vector Labs) solution and diaminobenzidine. For immunofluorescence studies, the secondary antibody was biotinylated anti-mouse, followed by streptavidin-linked Cy3.

### Antibodies

Monoclonal antibodies (antigen in parenthesis) were obtained from the Developmental Studies Hybridoma Bank (University of Iowa, USA): 3A10 (neurofilament-associated protein); 39.4D5 (Islet 1/2; detects both Isl1 & Isl2); 4F2 (detects both Lim1 and Lim2); 74.5A5 (Nkx2.2); 81.5C10 (MNR2/HB9; detects both MNR2 and HB9); 67.4E12 (Lim3); F55A10 (Nkx6.1); Pax6; Pax3; anti-BrdU. Antibodies were also used against phosphohistone (PH3[ser10], Upstate Biotechnology, Millipore UK), activated caspase-3 (Upstate Biotechnology, Millipore UK), phosphotyrosine (4G10; Millipore UK), β-catenin (6F9; Sigma Aldrich) and FLAG (M2; Sigma Aldrich UK). The polyclonal anti-avian PTPγ serum AB69 was raised using chick PTPγ peptide YQELQLDGFDNESSNKTWMK (aa99-118); The peptide and the antiserum were generated by Qbiogene (Nottingham, UK). DAKO UK Ltd. (Ely) supplied anti-mouse sera (raised in rabbit) conjugated to TRITC, FITC, HRP and biotin, as well as anti-guinea pig and anti-rabbit HRP-conjugates and goat anti-rabbit conjugated to biotin. Cy3 conjugated to streptavidin was obtained from GE Healthcare (Chalfont St.Giles, UK).

### In situ hybridisation

The chick PTPγ probe was generated as described (Ledig et al., 1999). The probe covered bp 2799–3144 of the cDNA (accession U38349) and has been shown to be specific (Chilton and Stoker, 2000; Ledig et al., 1999). RNA probes were synthesized according to manufacturer's protocols, using DIG-labelling kit (Roche Diagnostics, Burgess Hill, UK). Probes were denatured, hybridised overnight at 70°C and slides were then washed in 1x SSC, 50% Formamide, 0.1% Tween20 at 65°C followed by TBST. Slides were treated with anti-DIG antibodies according to manufacturer's protocols (Roche Diagnostics), followed by alkaline-phosphatase (AP)-linked secondary and standard AP detection. The chick NeuroM probe was provided by Marc Ballivet (Roztocil et al., 1997).

### Cell counts and GFP quantitation

Labelled neurons were counted on each side of individual spinal cords and a ratio of the numbers was made, using at least two sections per embryo. Means and standard deviations of these ratios were calculated for each treatment group. PH-3-positive cells were counted in the electroporated and contralateral ventricular zones in individual embryos and a ratio per embryo calculated; ventricular zone length was reduced by less than 5% by electroporation. Means and standard deviations of these ratios were then calculated. For quantitation of TOP-GFP expression, the total pixel intensities of co-electroporated RFP (from pCAβ-RFP) and GFP (from TOP-GFP) were measured in the top 60% of each spinal cord section, using Volocity software (Perkin Elmer/Improvision); 2 sections were measured for each embryo and a mean value taken. After background removal, the final GFP/RFP ratio was made for each embryo. In all studies, a Student *T*-test was carried out to calculate statistical significance. Measurement of nuclear orientation was carried out using Openlab. Spinal cord sections were stained with DAPI and 25–50 nuclei per spinal cord side were measured from individual embryos. Means and standard deviations were calculated and a ratio of SD generated for each embryo.

### Phosphatase enzyme assay

A purified form of the human PTPγ D1D2 domain was generated as previously described (Barr et al., 2009a). 293T HEK cells were treated for 15 min at 37 °C with 100 μM freshly prepared sodium peroxovanadate, before lysis in 0.25% TritonX-100, 150 mM NaCl, TrisHCl pH 7.6 with protease inhibitors (Complete, Roche), plus 1 mM sodium vanadate. β-catenin was immunopurified from lysates using 6F9 (Sigma Aldrich UK) and Protein G agarose (Roche). Purified PTPγ D1D2 was then mixed with 10 μl of agarose beads in phosphatase buffer (100 mM Hepes pH7.2, 2 mM EDTA, 2nM DTT, 0.1% NP40, 1 mg/ml BSA) and placed at 37 °C for 15 min with occasional agitation. The reaction was stopped by addition of gel loading buffer containing β-mercaptoethanol. Proteins were subjected to PAGE and immunoblotted using 4G10 anti-phosphotyrosine (Millipore, UK) Duplicate blots were probed with anti-actin (Sigma Aldrich, UK).

Supplementary materials related to this article can be found online at doi:10.1016/j.mcn.2010.11.012
